# Patterns and Predictors of Language and Literacy Abilities 4-10 Years in the Longitudinal Study of Australian Children

**DOI:** 10.1371/journal.pone.0135612

**Published:** 2015-09-09

**Authors:** Stephen R. Zubrick, Catherine L. Taylor, Daniel Christensen

**Affiliations:** Telethon Kids Institute, University of Western Australia, Perth, Western Australia, Australia; Kyoto University, JAPAN

## Abstract

**Aims:**

Oral language is the foundation of literacy. Naturally, policies and practices to promote children’s literacy begin in early childhood and have a strong focus on developing children’s oral language, especially for children with known risk factors for low language ability. The underlying assumption is that children’s progress along the oral to literate continuum is stable and predictable, such that low language ability foretells low literacy ability. This study investigated patterns and predictors of children’s oral language and literacy abilities at 4, 6, 8 and 10 years. The study sample comprised 2,316 to 2,792 children from the first nationally representative Longitudinal Study of Australian Children (LSAC). Six developmental patterns were observed, a stable middle-high pattern, a stable low pattern, an improving pattern, a declining pattern, a fluctuating low pattern, and a fluctuating middle-high pattern. Most children (69%) fit a stable middle-high pattern. By contrast, less than 1% of children fit a stable low pattern. These results challenged the view that children’s progress along the oral to literate continuum is stable and predictable.

**Findings:**

Multivariate logistic regression was used to investigate risks for low literacy ability at 10 years and sensitivity-specificity analysis was used to examine the predictive utility of the multivariate model. Predictors were modelled as risk variables with the lowest level of risk as the reference category. In the multivariate model, substantial risks for low literacy ability at 10 years, in order of descending magnitude, were: low school readiness, Aboriginal and/or Torres Strait Islander status and low language ability at 8 years. Moderate risks were high temperamental reactivity, low language ability at 4 years, and low language ability at 6 years. The following risk factors were not statistically significant in the multivariate model: Low maternal consistency, low family income, health care card, child not read to at home, maternal smoking, maternal education, family structure, temperamental persistence, and socio-economic area disadvantage. The results of the sensitivity-specificity analysis showed that a well-fitted multivariate model featuring risks of substantive magnitude did not do particularly well in predicting low literacy ability at 10 years.

## Introduction

Children’s language development builds the foundation for literacy, educational achievement and employment [1, 2]. Literacy is recognised as a human right and a means for achieving other human rights. The benefits that literacy confers on individuals, families, communities and nations are human, social, economic, cultural and political [3]. Speaking and listening are developmental prerequisites for reading, writing and spelling. This relationship entails dynamic complementarity and self-productivity which produce multiplier effects whereby language enables literacy and in turn, literacy enables language. This is especially true for vocabulary where in the beginning stages of reading, children rely heavily on their vocabulary knowledge to understand what they read, and once proficient, acquire new vocabulary through reading [4, 5]. Vocabulary knowledge is a strong predictor of reading comprehension [6] and poor vocabulary knowledge is associated with low literacy achievement [7]. Population based prospective longitudinal studies have documented the enduring association between low language ability in the preschool years and low literacy in childhood and adulthood [1, 8–10].

Improving language and literacy standards is an important global [11] and national goal [12]. There is unanimous agreement that the policy focus must begin early in childhood, before the start of formal school and formal reading instruction [10, 13, 14]. In the preschool years there is a strong focus on developing children’s oral language abilities, especially for children with known risk factors for low language ability.

The predominant theory about reading, “The Simple View of Reading,” was first advanced by Gough and Tunmer [15] and is prominent in early childhood education policies and pedagogies [10]. This early view breaks down reading into two dimensions, decoding the alphabetic system, which requires code-related abilities, and, understanding the written word, which requires oral language abilities. Code-related abilities include knowledge of letter names and letter sounds, phonological awareness and writing. Oral language abilities include vocabulary, grammar and listening comprehension[16]. These components of oral language are interdependent but vocabulary is considered to be the “lynchpin”. The association between children’s vocabulary and literacy abilities is most closely associated at the beginning stages of learning to read, yet it is still evident years later in adulthood [1, 8–10].

Effective early language and literacy intervention promotes children’s code-related and oral language abilities [16]. Decoding and comprehension are both necessary for proficient reading. Poor readers can be deficient in both decoding and comprehension or in only one component of reading. Some poor readers have strong decoding ability and poor comprehension while others have poor decoding ability and strong comprehension [17].

Most longitudinal studies of literacy outcomes for children and adults with a history of low language ability have been designed to quantify the persistence of low abilities over time [3, 18, 19]. There has been an understandable interest in the developmental pathways that lead towards low language ability. A consequence of this focus has been the relative neglect of the question of instability or change in language ability over time—the extent of this, and, its implications for developmental research and practise.

On close inspection, it is possible to find studies of evidence of change of literacy ability over time. An early study of Phillips et al [20] noted marked variability and associated poor prediction in reading and academic performances of children in Year 1 to Year 6 of school. Catts et al [7] in a population based study of reading outcomes for children aged 5–6 years with specific language impairment estimated 42% of these children had a reading impairment in first grade yet 58% did not. Notably, 9% of children with typical language development went on to have reading impairment in first grade. The prevalence of reading impairment in the children with language impairment decreased from 42% to 36% in fourth grade, evidence of changing developmental status over time for some children. Additionally, 30% of the children with specific language impairment had reading impairment in one grade but not the other. Certainly the common view is that children progressively acquire and stabilise skills in the early childhood epoch. But the Catts et al. findings, point towards more instability in literacy performances than might be expected in children over time.

These observations of instability in the emergence and establishment of literacy have a parallel in recent studies of early vocabulary acquisition. Taylor et al. [21] and Christensen et al [22] documented vocabulary growth trajectories and modelled multivariate predictors of vocabulary performance in a large-sample population longitudinal study of 4 to 8 year olds. They documented both marked variability in initial vocabulary levels and in onward growth with resultant poor positive prediction of those 4 year old children who would go on at age 8 to have low vocabulary. Of 1,083 children predicted to have low vocabulary at age 8, only 279 (25.8%) actually had low vocabulary. The strongest predictor of low receptive vocabulary at age 8 was low receptive vocabulary at age 4. However, receptive vocabulary was nonetheless a limited predictor of persistently low receptive vocabulary despite its moderate odds ratio with vocabulary ability at 8.

Given the documented association between early vocabulary development and subsequent reading ability we investigate here the onward strength of this association and its predictive utility. Specifically we investigate the uncertainty about the early developmental variability in onward language and literacy and whether this reduces and converges as children age and acquire skills. To do this we use a large representative sample of Australian children to estimate the longitudinal predictive relationship between vocabulary status at ages 4, 6 and 8 years and onward literacy performance at age 10. We focus on three questions: 1) What are the patterns of stability and change in children’s progression from early vocabulary development to their onward literacy; 2) what are the predictors of this progression, and; 3) what is the predictive utility of this relationship?

## Methods

### Ethics statement

The Longitudinal Study of Australian Children (LSAC) is conducted in a partnership between the Department of Social Services (DSS), the Australian Institute of Family Studies (AIFS) and the Australian Bureau of Statistics (ABS). The study has ethics approval from the Australian Institute of Family Studies Ethics Committee. The Ethics Committee is registered with the Australian Health Ethics Committee, a subcommittee of the National Health and Medical Research Council (NHMRC). Caregivers gave written informed consent to the survey. As the study children were all minors at the time these data were collected, written informed consent was obtained from the caregiver on behalf of each of the study children. The signed consent forms are retained by the field agency (ABS).

### Access and use of LSAC data

Confidentialised LSAC data are publicly available. Researchers can apply to the Commonwealth of Australia Department of Social Services (DSS) for permission to access and use Longitudinal Study of Australian Children (LSAC) data (Growing up in Australia website http://www.growingupinaustralia.gov.au/data/dataaccess.html Accessed 29 June 2015).

### Study design

The initial study sample comprised 2,792 children who participated in the Longitudinal Study of Australian Children (LSAC), and had data for the key variables (language and literacy) at each wave of the survey. The analytic sample was less than the wave 4 sample, as not all children responded to all study items. The sample size thus varied slightly between analyses, based on item response.

The LSAC is a national longitudinal study that commenced in 2004. The study uses a cross-sequential design of biennial face-to-face visits with the family and study child. In this study we used data from the child cohort collected at 4, 6, 8 and 10 years. Table 1 contains the number of children, the median ages and age ranges in months for the study children at each longitudinal wave.

**Table 1:** Sample size at each wave, children’s ages, and PPVT and ARS scores with available sample.

	Sample at each wave			Measures			
Wave	N	Child’s age in months			N^1^	Mean (SD)	Range
		Median	Range	Measure			
1	4983	57	51–67	PPVT	4406	65 (6.0)	28–85
2	4464	82	75–94	PPVT	4317	74 (5.0)	46–92
3	4331	105	95–119	PPVT	4273	78 (5.0)	45–106
4	4169	130	121–140	ARS	3336	3.79 (0.9)	1–5

^1^Available sample size.

The LSAC sampling frame was extracted from the Medicare Australia enrolment database, which was validated to ensure coverage of Australian children within the target age-range. The initial study sample was designed to be representative of Australian children within the selected age cohort, proportional to the regional distribution of children in the Australian population. An initial sample size of 5,000 was chosen as to ensure there would still be a sufficient sample for detailed analysis after attrition over the number of years of the longitudinal study.

The study entailed a two-stage clustered design, first selecting postcodes then children within postcodes. Stratification was used to ensure proportional geographic representation for states/territories and capital city statistical division/rest of state areas. Cluster sampling was utilised because it provides a cost effective way to conduct face-to-face interviews, as well as an opportunity to collect and analyse community-level effects. Postcodes were selected with probability proportional to size selection where possible, and with equal probability for small population postcodes. Children were selected from 311 postcodes [23, 24].

Analyses show that the initial sample was broadly representative of the general Australian population when compared with 2001 Census data, but slightly under-representative of families who were single-parent, non-English speaking and living in rental properties [25]. Attrition somewhat increased these biases. For example, the overall attrition rate between ages 4 and 10 was 16.3%, but children with mothers classified as Non-English speaking background decreased from 15.6% at age 4 to 13.3% at age 10, an attrition rate of 28.9%. The proportion of mothers who had a year 11 or less education decreased from 38.9% at age 4 to 35.9% at age10, an attrition rate of 22.7%.

### Measures

#### Measures of language and literacy abilities

The measure of language ability was the Adapted Peabody Picture Vocabulary Test-III (PPVT) and the measure of literacy ability was the Academic Rating Scale: Language and Literacy Subscale (ARS).

The Adapted Peabody Picture Vocabulary Test-III (PPVT) is a test of receptive vocabulary designed for the LSAC study [26]. The Adapted PPVT-III is a shortened version of the PPVT–III [27]. The Adapted PPVT-III was administered directly to each child during the home interview. For each word presented, the child was shown a card containing four pictures and was asked to point to the picture corresponding to the word (e.g., “Show me wrapping”). Scaled scores for the Adapted PPVT-III were used in all analyses. The Pearson product-moment correlation between the full PPVT-III and the Adapted PPVT-III was 0.93 for all children [26].

The Academic Rating Scale: Language and Literacy Subscale (ARS) is a teacher-report measure of literacy ability that has been demonstrated to validly assess developmental skill levels consistent with theories of early literacy acquisition [28]. The ARS comprises nine items (e.g. conveys ideas when speaking, uses strategies to gain information from print, reads fluently, reads grade-level books, comprehends informational text, composes multi-paragraph texts, redrafts, writing, makes editorial corrections, uses computer for variety of purposes). Teachers rated the study child’s performance on a five-point ordinal scale (1 = ‘not yet’; 2 = ‘beginning’; 3 = ‘in progress’; 4 = ‘intermediate’; 5 = ‘proficient.’) in relation to other children at the same grade level. These ratings were used to produce a composite score via Rasch modelling. The development of this measure is described extensively elsewhere [29–31].

Table 1 contains the mean scores with SDs and associated ranges for the Rasch scaled PPVT and ARS.

#### Age standardising

In this study we wanted to examine the relationship between language and literacy over time. However, previous work by Taylor et al [21] has established the relationship between child age and receptive vocabulary. A linear regression model established a slope of 0.35 at age 4. That is, for each month of advancing age, we expect an increase of 0.35 in their PPVT scores. Therefore, in preparing the data for analyses, we decided to adjust for child age, by comparing children’s receptive vocabulary and literacy to their peers closest to them in age.

To age standardise, we created age-groups of approximately equal size within the 4-year, 6-year, 8-year, and 10-year age-groups (see Table 2). Children in these age-groups were then ranked relative to their peers. This also allowed us to compare PPVT and ARS on the same scale, as we are now assessing children’s language and literacy relative to similar-aged peers.

**Table 2:** Age-groups within the 4-year, 6-year 8-year, and 10-year age-groups.

4-year age-group		6-year age-group		8-year age group		10-year age-group	
age-group months	n	age-group months	n	age-group months	n	age-group months	n
51–54	940	75–79	943	95–102	670	121–126	726
55–56	1292	80–81	1101	103–104	921	127–129	1241
57–58	1363	82–83	1116	105–106	1160	130–131	853
59–60	943	84–85	821	107–108	846	132–134	886
61–67	445	86–94	483	109–119	734	135–140	463
**Total**	**4983**	**Total**	**4464**	**Total**	**4331**	**Total**	**4169**

#### Stability and change patterns

**Fig 1:**
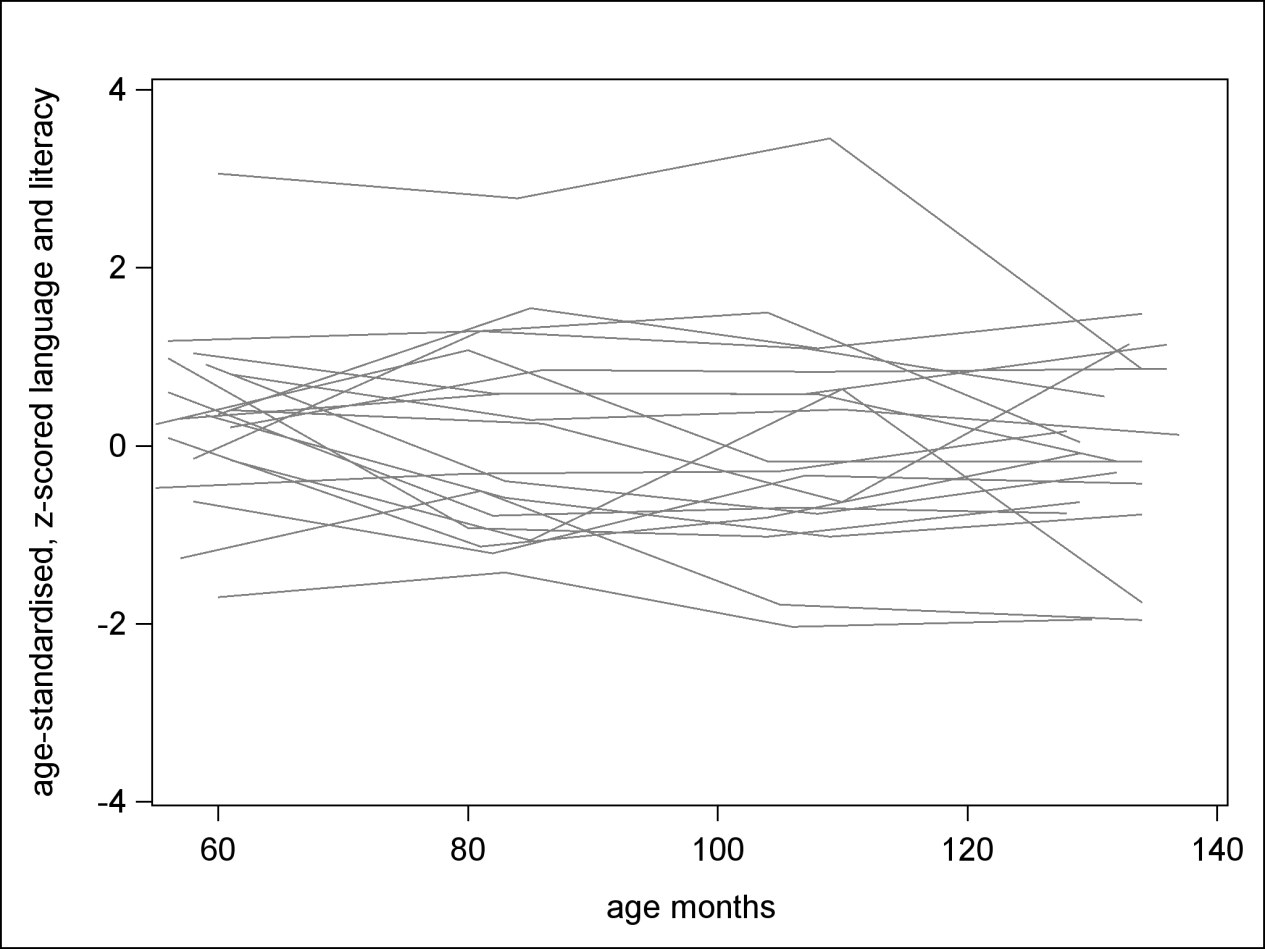
Variation in language and literacy at ages 4, 6, 8 and 10.

Fig 1 shows a sample of age-standardised PPVT and ARS z-scores for 20 children randomly selected from the LSAC. It shows that children’s language ability exhibits considerable variability over time. A child that starts well above the mean in PPVT standard deviation units at age 4 does not necessarily finish with ARS well above the mean in standard deviation units at age 10. Age-standardised z-scores permit assessing the magnitude of the predictive association between PPVT at age 4 and ARS at age 10 and estimation of the contribution of other putative predictors of teacher-rated literacy.

Finally, as we were interested in stability and change in receptive vocabulary and literacy, we identified the lowest 15% (‘low’) within each age-group. This enabled us to examine the extent to which being low-performing in receptive vocabulary, relative to their peers at ages 4, 6 and 8 years, is predictive of their onward teacher-rated language and literacy at 10 years. Taking a categorical approach to language and literacy is complementary to growth curve modelling [21]. It allows us to establish the likelihood of children being low-performing relative to their peers at age 10, and it allows us to establish how well low literacy ability at age 10 can be predicted from risk factors measured at age 4. The latter question is one of substantial practical importance for policies and resources for screening, intervention and progress monitoring.

**Fig 2:**
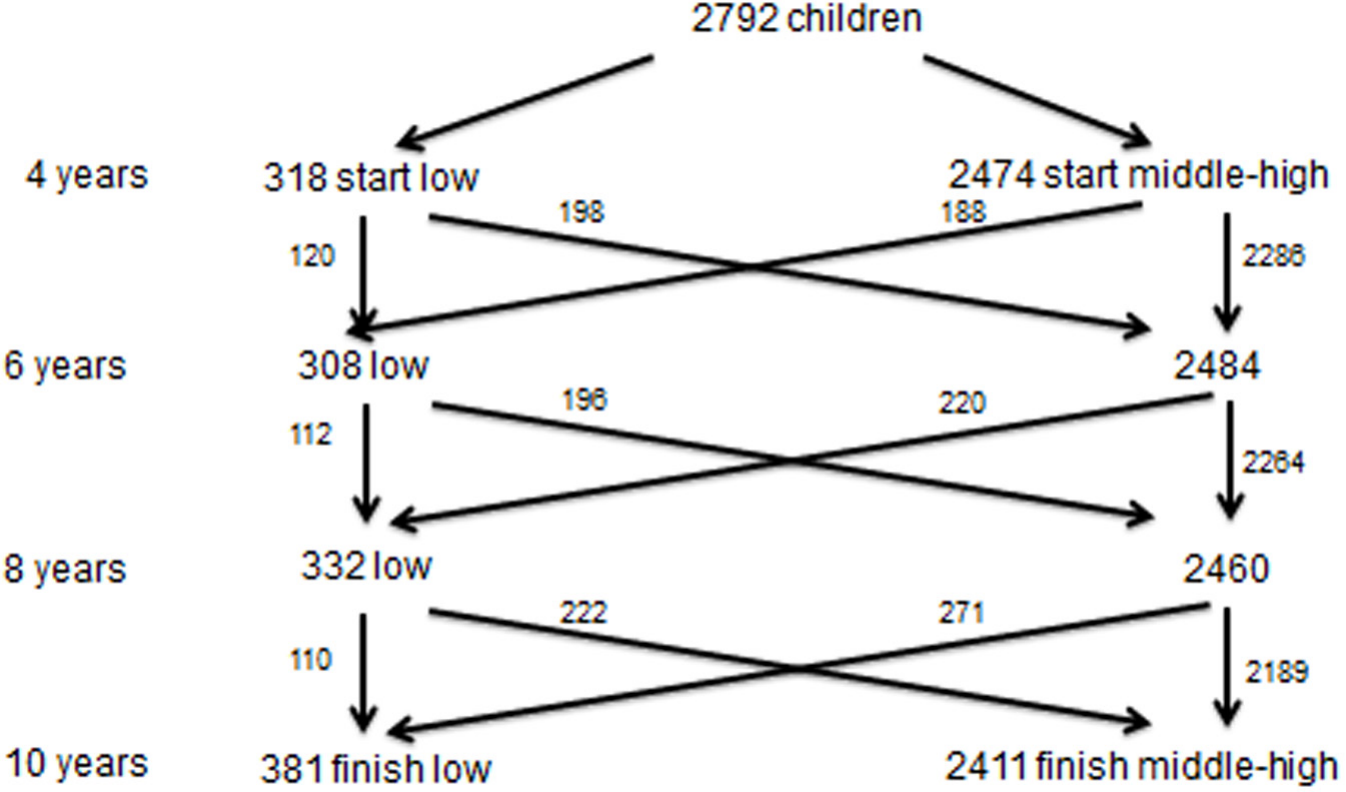
Positional movement in language and literacy at ages 4, 6, 8 and 10.

Fig 2 illustrates movement between groups at each age for those children where there is language data recorded at all 4 ages (n = 2792). These can be summarised as patterns of stability, change, improvement and decline. Fig 2 shows that of 318 children with low language at age 4, more than half (198, 62%) move to the middle-high group at age 6.

What Fig 2 does not show is specific grouping of children in patterns of stability and change. If we consider that a child can be grouped as either ‘low’ or ‘middle-high’ at ages 4, 6, 8 and 10,there are 16 (2^4^) possible combinations of language development across time. Table 3 illustrates these combinations.

**Table 3:** Language performance within the 4-year, 6-year 8-year, and 10-year age-groups.

Age 4	Age 6	Age 8	Age 10	n	%	Pattern
Low	Low	Low	Low	26	0.9	Stable low
Low	Low	Low	Middle-High	27	1.0	Improving pattern
Low	Low	Middle-High	Low	17	0.6	Fluctuating low pattern
Low	Low	Middle-High	Middle-High	50	1.8	Improving pattern
Low	Middle-High	Low	Low	21	0.8	Fluctuating low pattern
Low	Middle-High	Low	Middle-High	30	1.1	Improving pattern
Low	Middle-High	Middle-High	Low	29	1.0	Fluctuating low pattern
Low	Middle-High	Middle-High	Middle-High	118	4.2	Improving pattern
Middle-High	Low	Low	Low	20	0.7	Declining pattern
Middle-High	Low	Low	Middle-High	39	1.4	Fluctuating middle-high pattern
Middle-High	Low	Middle-High	Low	23	0.8	Declining pattern
Middle-High	Low	Middle-High	Middle-High	106	3.8	Fluctuating middle-high pattern
Middle-High	Middle-High	Low	Low	43	1.5	Declining pattern
Middle-High	Middle-High	Low	Middle-High	126	4.5	Fluctuating middle-high pattern
Middle-High	Middle-High	Middle-High	Low	202	7.2	Declining pattern
Middle-High	Middle-High	Middle-High	Middle-High	1915	68.6	Stable middle-high
**Total**				**2792**	**100**	

These groupings of language development can be split into six broad patterns: 2 patterns of stability and 4 patterns of change. Broadly, children can either be stable in their language (i.e. the same position at each age) or they can change (i.e. they change position at least once between ages). The two stable patterns were a stable middle-high pattern (i.e., middle-high across all 4 ages) and a stable low pattern (i.e., low across all 4 ages). The most common developmental pattern was the stable middle-high pattern, 69% of the children were middle-high at all 4 ages. One of the least common developmental patterns was a stable low pattern. Only 26 children (less than 1% of all children) were persistently in the low group at ages 4, 6, 8 and 10.

Change patterns are also evident in Table 3. Children can show an improving pattern (i.e., starting low and finishing middle-high), a declining pattern (starting middle-high and finishing low), a pattern of low fluctuation (i.e. starting low, moving to middle-high, then finishing low), or a pattern of middle-high fluctuation (i.e. starting middle-high, moving to low, then finishing middle-high). Eight per cent (8%) of children fit an improving pattern, 10% of children fit a declining pattern, 2% of children are in a fluctuating low pattern, and 10% of children are in a fluctuating middle-high pattern. Of the 318 children who started in the low language group at age 4, only 93 finished in the low group at age 10. Of the 381 children who were low in language at age 10, the majority (355) were classified as middle-high at least once at ages 4, 6 and 8. The table illustrates some strong trends of instability. For example, 637 children out of 2792 spend 3 waves in one group and 1 wave in another group (i.e. low, low, low, middle-high, middle-high; low, low, low; low, middle-high, low, low…).

#### Candidate predictors

The candidate predictor measures used in this paper have been extensively described elsewhere [3].

Briefly, a bioecological model of child development [32, 33] guided the selection of measures for the LSAC. Among these domains are characteristics related to the child, the mother, and the family home environment. In this study, we used the same predictor set used in our study of receptive vocabulary growth 4–8 years [21]. Variables in this predictor set met one of the following two criteria: (1) Evidence of an independent association with English language abilities in a representative population level sample of preschool and school age children; or (2) conceptual relevance to language abilities, in the absence of empirical evidence. Many of the measures are benchmarked against Australian census collections while others are referenced to large scale Australian and international child development studies.

In addition to measures of PPVT at ages 4, 6 and 8, another 28 candidate predictor variables were used, giving a total of 31 predictors. These were grouped into child, maternal, and family and home environment characteristics. All candidate predictor variables were measured when the child was 4 years of age. Candidate predictors were modelled as risk variables with the lowest level of risk as the reference category (see Table 4). The analytic sample for each candidate predictor varied somewhat, depending on item completeness.

**Table 4:** Initial candidate predictors: Child, maternal and family characteristics of children in the low literacy and middle-high literacy groups at 10 years.

Predictor variables	Low ARS at 10 years (N = 502)		Middle-high ARS at 10 years (N = 2834)		Unadjusted Odds Ratio^a^ ^b^		p
	n	%	n	%	OR	95%CI	
**PPVT group at age 4**							<.0001
Low (bottom 15%)	114	31.6	247	68.4	3.43	2.62–4.49	
Middle-high group	312	11.7	2351	88.3	ref.		
**PPVT group at age 6**							<.0001
Low (bottom 15%)	117	30.8	263	69.2	3.07	2.47–3.82	
Middle-high group	343	12.5	2406	87.5	ref.		
**PPVT group at age 8**							<.0001
Low (bottom 15%)	145	34.3	278	65.7	3.72	2.96–4.69	
Middle-high group	325	11.6	2478	88.4	ref.		
**Child Characteristics at age 4**							
**Sex**							<.0001
male	321	19	1369	81	1.75	1.44–2.12	
female	181	11	1465	89	ref.		
**Ethnicity**							<.0001
ATSI^C^	45	45	55	55	4.92	3.23–7.48	
SC Non-ATSI	457	14.1	2777	85.9	ref.		
**Birthweight**							0.1053
Low birthweight	38	18.3	170	81.7	1.34	0.94–1.89	
Normal birthweight	450	14.6	2638	85.4	ref.		
**SC Ear Infections**							0.0005
yes	59	23	197	77	1.69	1.26–2.27	
no	443	14.4	2637	85.6	ref.		
**Who Am I (quintiles)**							<.0001
1 (lowest)	205	34	398	66	11.16	7.17–17.38	
2	135	18.9	579	81.1	5.36	3.45–8.32	
3	63	11.5	487	88.5	2.93	1.86–4.61	
4	52	7.7	627	92.3	1.59	0.98–2.58	
5 (highest)	32	4.2	722	95.8	ref.		
**Persistence (quintiles)**							<.0001
1 (lowest)	118	24.2	369	75.8	3.59	2.51–5.13	
2	128	16.6	645	83.4	2.16	1.49–3.13	
3	82	11.8	610	88.2	1.51	1.04–2.17	
4	55	10.7	461	89.3	1.4	0.93–2.09	
5 (highest)	37	8	423	92	ref.		
**Reactivity (quintiles)**							<.0001
1 (most reactive)	131	22	464	78	2.73	1.92–3.88	
2	82	16.1	427	83.9	1.81	1.27–2.57	
3	78	12.6	542	87.4	1.38	0.99–1.93	
4	75	12.4	531	87.6	1.29	0.89–1.88	
5	50	8.6	532	91.4	ref.		
**Sociability (quintiles)**							0.8966
1 (lowest)	77	14.7	446	85.3	0.92	0.67–1.26	
2	109	14.9	622	85.1	1.05	0.77–1.44	
3	86	14.1	524	85.9	0.92	0.67–1.27	
4	71	12.2	510	87.8	0.92	0.65–1.29	
5	76	15.6	410	84.4	ref.		
**Maternal Characteristics at age 4**							
**Mother’s age at birth**							0.4385
teen	17	22.4	59	77.6	1.43	0.81–2.52	
40 and over	12	12.5	84	87.5	0.87	0.47–1.62	
20–39	462	14.8	2666	85.2	ref.		
**Mother alcohol problem**							0.0131
yes	60	17.5	282	82.5	1.46	1.08–1.96	
no	346	13.7	2176	86.3	ref.		
**Mother smoker**							<.0001
yes	133	21.4	488	78.6	2.06	1.64–2.6	
no	286	12.4	2025	87.6	ref.		
**Mother K6 symptomatic**							<.0001
yes	90	21.2	335	78.8	1.81	1.4–2.34	
no	325	13.1	2165	86.9	ref.		
**Maternal education**							<.0001
Year 12	149	13.7	940	86.3	1.68	1.29–2.19	
Year 11 or less	266	22.4	920	77.6	3.25	2.53–4.17	
University	83	8	959	92	ref.		
**Maternal work hours**							<.0001
zero hours (includes not in labour force)	249	18.5	1095	81.5	1.86	1.5–2.3	
full-time: 38 hours+	62	14	381	86	1.08	0.79–1.48	
part time: 1–37 hours	191	12.3	1358	87.7	ref.		
**Maternal consistency (quintiles)**							<.0001
1 (least consistent)	156	24.6	478	75.4	3.25	2.44–4.33	
2	94	18.1	426	81.9	2.09	1.53–2.86	
3	89	11.9	656	88.1	1.27	0.91–1.76	
4	86	12.1	623	87.9	1.22	0.88–1.7	
5	70	10.1	622	89.9	ref.		
**Maternal inductive reasoning (quartiles)**							0.8426
1 (lowest reasoning)	94	17.7	437	82.3	1.08	0.81–1.43	
2	159	13.8	996	86.2	0.95	0.74–1.22	
3	116	15.2	647	84.8	0.98	0.76–1.27	
4	126	14.8	726	85.2	ref.		
**Maternal warmth (quintiles)**							0.0319
1 (lowest warmth)	114	15.9	604	84.1	0.84	0.62–1.12	
2	111	13.4	720	86.6	0.68	0.51–0.9	
3	64	15.9	339	84.1	0.79	0.57–1.08	
4	99	13.2	650	86.8	0.65	0.48–0.88	
5	108	18	493	82	ref.		
**Maternal hostility (quintiles)**							0.0007
1 (greatest hostility)	134	18.7	584	81.3	1.69	1.26–2.25	
2	128	14.2	774	85.8	1.28	0.94–1.75	
3	69	13	463	87	1	0.72–1.4	
4	102	16.8	504	83.2	1.53	1.1–2.11	
5	63	11.6	480	88.4	ref.		
**Family characteristics at age 4**							
**Mother Non-English speaking background**							0.0673
yes	46	11.6	350	88.4	0.76	0.56–1.02	
no	452	15.5	2470	84.5	ref.		
**Family structure**							<.0001
Single mother family	96	27.7	251	72.3	2.41	1.88–3.09	
Other	406	13.6	2583	86.4	ref.		
**Siblings**							<.0001
1 sibling	208	12.4	1467	87.6	0.58	0.43–0.77	
2 siblings	138	15.1	776	84.9	0.73	0.53–0.99	
3 siblings	65	22.1	229	77.9	1.28	0.86–1.89	
4 or more siblings	27	26.2	76	73.8	1.61	0.96–2.69	
only child	64	18.3	286	81.7	ref.		
**Family income**							<.0001
under $600/week	118	26.6	326	73.4	4.1	2.88–5.82	
$600–999	130	17.9	596	82.1	2.45	1.75–3.43	
$1,000-$1,499	108	13.2	709	86.8	1.7	1.19–2.44	
$1,500-$1,999	65	11	526	89	1.28	0.88–1.85	
$2,000 or more/week	52	9	523	91	ref.		
**Health care card**							<.0001
yes	160	26.1	452	73.9	2.46	1.99–3.05	
no	342	12.6	2382	87.4	ref.		
**Financial hardship**							<.0001
yes	200	21.8	718	78.2	1.87	1.53–2.29	
no	302	12.5	2113	87.5	ref.		
**SEIFA disadvantage index (quintiles)**							<.0001
1 (lowest SEIFA)	146	21.5	534	78.5	2.34	1.7–3.23	
2	116	16.4	591	83.6	1.52	1.11–2.08	
3	87	13.3	567	86.7	1.24	0.88–1.75	
4	77	12.3	550	87.7	1.16	0.82–1.64	
5	76	11.4	592	88.6	ref.		
**Reads to child**							<.0001
no reading	26	29.5	62	70.5	3.48	2.21–5.45	
1–2 days/ week	123	20.6	475	79.4	2.15	1.68–2.76	
3–5 days/ week	170	16.7	848	83.3	1.59	1.25–2.01	
daily	182	11.2	1448	88.8	ref.		
**Playgroup**							0.1240
no	298	15.1	1675	84.9	1.19	0.95–1.49	
yes	121	12.4	852	87.6	ref.		
**Hours a week in care**							0.1141
9–20	330	15.4	1806	84.6	1.48	0.92–2.4	
21–30	83	13.1	550	86.9	1.14	0.68–1.91	
31+	41	15.9	217	84.1	1.48	0.82–2.65	
0–8	22	11.1	177	88.9	ref.		

^a^Weighted

^b^95% Confidence Intervals

^c^Study child Aboriginal and/or Torres Strait Islander status

#### Child characteristics

The child characteristics in our models were: Gender, ethnicity, birthweight, ear infections, school readiness and temperament. There were equal proportions of girls and boys in the sample. A small proportion of children (n = 100; 3.0%) were of Aboriginal and/or Torres Strait Islander descent and were coded to distinguish them from those who were not. Primary carers were asked to report their child’s birthweight which was subsequently coded into those children who were born with low birthweight (< 2500 grams; 6.3%) and those who weighed more than this (> = 2500 grams). A single item indicator of ongoing ear infections at 4 years was included.

Each study child was directly assessed at 4 years using the *Who Am I*?*(WAI)* [34]. The 11 items of the WAI measure ‘pre-academic’ early copying and writing skills. The items include visuo-spatial, manual-motor copying of shapes (5 items), a single item that probes for the child’s capacity to write a number(s), a single item prompting the child to draw a person (picture of yourself), and four items that probe literacy: write some letter(s), your name, some word(s) and a sentence. The WAI is broadly independent of language background, and is considered a general measure of aspects of early literacy and motor control (see Prior et al., 2013 [35]). It has been extensively calibrated for use in the LSAC and has well demonstrated item characteristics, high internal reliability (0.89), and excellent distributional properties [36]. There is a weak correlation between the WAI and PPVT measure at age 4: About 9% of their variance is shared [36]. In this report, study children have been grouped into quintiles of performance based on the total *Who Am I*? score with high quintiles representing higher levels of performance.

Child temperament was measured at 4 years with the Short Temperament Scale for Children (STSC) [37]. The STSC measures three dimensions of temperament: persistence, reactivity and sociability. Items for each measure were summed to create a composite score, and each composite was then divided into quintiles with higher quintiles representing the positive aspects of each dimension.

#### Maternal characteristics

The maternal characteristics in our models were: Age at the birth of the child, problematic alcohol use, smoking, mental health distress, education, hours of paid employment and parenting. The biological mother’s age at the birth of the child was grouped into with the vast majority of mothers (94.8%) of study children in the age range 20–39 years.

Information on current tobacco and alcohol was gathered from the mothers. We defined problematic alcohol use where women reported their daily alcohol consumption to exceed 2 standard drinks and/or where they reported frequent binge drinking of 5 or more alcoholic drinks at least 2–3 times per month. Study children’s mothers were asked about tobacco use and also categorised as either current smokers (21.2%) or not current smokers.

In this study, we used the Kessler-6 (K6) scale to measure maternal non-specific psychological distress. Women with scores of 8 or more were classified as having symptomatic psychological distress. This threshold is consistent with other studies [38–40] using the K6. About fifteen per cent of mothers reported symptomatic psychological distress.

In Australia, at the time of this study, 10 years of education was compulsorily mandated. Maternal education in years was grouped into three levels according to those who had completed 11 years or less (35.8%), 12 years (32.8%), and those who had completed more than 12 years (i.e. University education) (31.4%).

We used total hours of paid maternal employment to distinguish mothers who were not in paid employment (0 hours), from those in part time paid employment (1–37 hours; Median = 18 hours) and in full time paid employment (> = 38 hours; Median = 44 hours). Similar proportions of women were either not in paid employment (40.3%) or working part time (46.4%) with the remainder (13.3%) working full time.

The parenting characteristics of both parents were measured in a self-complete form, using four measures of parenting warmth, hostility, consistency and inductive reasoning developed for the LSAC [41]. We use the mother’s responses in this report. Items for each measure were summed to create a composite score with higher levels representing more positive parenting characteristics. Item and scale properties for the LSAC parenting measures have been extensively documented [42].

#### Characteristics of the family home environment

The characteristics of the family home environment in our models were: maternal non-English speaking background (NESB), family structure, sibship size, income, health care card, financial hardship, socioeconomic disadvantage, reading to the study child, playgroup and child care. As the focus of this study is explicitly on English language development and because language development is known to vary where more than one language is spoken in the home, we used the mother’s NESB as a general indicator for language other than English spoken in the household at 4 years. About 12% of mothers were predominately non-English speaking at the time of the interview.

With respect to family composition, two variables were selected as candidate predictors of vocabulary development: Family structure (sole parent vs. other) and number of siblings (0, 1, 2, 3, 4+). About 10.4% of the study children were living in single mother families at 4 years. The majority had one or two siblings (50.2% and 27.4% respectively) or were singletons (10.5%) at age 4. The study questionnaire did not permit the establishment of birth order.

Families were asked to report their total weekly family income from all sources. Responses were partitioned into relatively equal quintiles. In Australia where income falls below a defined threshold and/or certain hardship criteria are met families also qualify for a health care card. About 18.3% of LSAC families had a health care card and this is used as an indicator of financial need in the LSAC families. Additionally, an indicator of family hardship was also derived where families reported, due to shortage of money over the last 12 months that: they had not been able to pay gas, electricity or telephone bills on time; they had not been able to pay the mortgage or rent on time; adults or children had gone without meals; they family had been unable to heat or cool their home; they had pawned or sold something; or sought assistance from a welfare or community organisation. About thirty per cent of families reported at least one of these occurrences in the previous 12 months at 4 years.

An area measure of socioeconomic disadvantage was also estimated for each participating family. The family home was coded with Socio-Economic Indicators for Area (SEIFA) disadvantage, indexed in quintiles—lower quintiles represent greater levels of disadvantage. The neighbourhood SEIFA disadvantage index summarises information from the Australian Census of Population and Housing as this relates to economic and social disadvantage in small areas, such as low income, low educational attainment and high unemployment [43]. These data were linked at the Statistical Local Area (SLA) level or, where this was not available, the child’s postcode.

Several indicators of the child’s learning environment were gathered. The frequency with which the primary caregiver read to the study child was assessed via face-to-face interview. A total of 88 (2.6%) of parents reported not reading to the child at all, 598 (17.9%) reported reading 1 or 2 days a week, 1,018 (30.5%) reported reading to the child 3–5 days a week, and 1,630 (48.9%) reported reading to the child daily. Mothers were asked if their child had attended a playgroup in the period 12 months prior to the 4-year interview with about one third indicating this to be the case. Finally, hours per week in child care or early education were identified by asking the primary carer “how many hours a week on average does the child go to school, kindergarten, pre-school, and/or day-care?” A total of 199 (6.2%) attended 8 or less hours a week, 2,136 (66.2%) attended 9–20 hours a week, 633 (19.6%) attended 21–30 hours a week, and 258 (8.0%) attended 31+ hours a week.

### Data analysis

The data were analysed using logistic regression in SAS 9.3 [44]. The surveylogistic procedure was used to account for the complex survey design of the LSAC. Logistic regression was used to estimate the odds of children being in the low group of ARS (lowest 15% in each age-group) at wave 4 of the LSAC (age 10–11 years).

Our analysis proceeded in four steps. First, we used logistic regressions to estimate initial unadjusted associations and effect sizes between our individual candidate predictors and ARS group (i.e., low vs. middle-high) at 10 years. Second, we grouped the candidate predictors by their unadjusted estimated effect size. Third, we set a criterion for selecting from these unadjusted effect sizes the final predictors to use in estimating a multivariate logistic regression model. Finally, we undertook a sensitivity-specificity analysis to examine the predictive utility of our multivariate model.

To establish the effect size for the selection of multivariate predictors in our final model we used the odds ratios (see Table 4) as established by the initial unadjusted logistic regressions. For logistic regression, the odds ratio itself represents the effect size of interest [45]. Although some schemes exist to estimate a correspondence between odds ratios and substantive effect sizes (e.g. [45, 46]), the judgement of what denotes a substantive effect size ultimately rests with the researcher, and must be considered within the context of the field of study [47]. For this paper we established an odds ratio of 2 or greater as our cut-off for a moderate effect size from which to draw the final set of predictors.

## Results

Table 4 shows the initial candidate child, maternal and family home environment predictor variables measured at age 4 and the percentage of the sample, at different levels of risk in the low ARS group versus the middle-high ARS group at 10 years. For example, of the 603 children who scored in the lowest quintile for school readiness (Who Am I?) at age 4, 34% were in the low ARS group at age 10. In contrast, of the 722 children in the most favourable quintile for school readiness (Who am I?) at age 4, only 4.2% were in the low ARS group at age 10. This corresponds to an odds ratio for the lowest performed WAI quintile 11.2 times greater than that for children in the highest performed quintile. Of note is that although children in the lowest WAI quintile were at 11.2 times the odds of children in the best performed quintile of ending up with low ARS at age 10, 66% of the children in the lowest-performed WAI quintile still went on to finish with middle-high ARS performances at age 10.

Of the 361 children who scored in the low PPVT group at age 4, 31.6% were in the low ARS group at age 10, while of the 2,663 children tested in the middle-high PPVT group at age 4 only 11.7% were in the low ARS group at age 10. This corresponds to an odds ratio for the low PPVT group, 3.4 times greater than that for children in the middle-high PPVT group. This latter observation is important. It illustrates that while low PPVT at age 4 carries with it an increased risk of low ARS at age 10, the majority of children with low PPVT at age 4 go on to achieve middle-high performances at age 10.

### Selection of predictors

Of the 31 initial unadjusted candidate predictors of low literacy (Table 4), fifteen were above our criterion odds ratio cut-off of 2.0 and were thus selected for multivariate modelling.

In order of increasing magnitude of effect size these were: Mother smoker (2.06), socio-economic area disadvantage (2.34), single-mother family (2.41), health care card (2.46), high reactivity (2.73), low PPVT score at 6 years (3.07), low maternal parenting consistency (3.25), low maternal education (3.25), low PPVT score at 4 years (3.43), child not read to at home (3.48), low persistence (3.59), low PPVT score at 8 years (3.75), low family income (4.10), Aboriginal and/or Torres Strait Islander status (4.92), and low WAI school readiness (11.16).

Nine candidate predictors of low literacy were statistically significant in the bivariate model, but fell below the OR 2.0 cut-off: 1 sibling vs. only child (0.58), problematic alcohol use (1.46), maternal hostility (1.56), ear infections (1.69), SC male (1.75), maternal mental health distress (1.81), mother not in work force (1.86), and financial hardship (1.87).

Eight candidate predictors of low literacy were not statistically significant in the bivariate model: daily use of non-parental child care, teenage mother status, low birthweight, not attending playgroup, low maternal inductive reasoning, low sociability, low maternal warmth and mother NESB.

### Multivariate analysis

The fifteen predictors of low literacy meeting the cut-off criterion of an odds ratio of 2.00 or higher in the unadjusted analyses were subsequently included in the multivariate model (see Table 5).

**Table 5:** Multivariate associations between child, maternal and family characteristics and low literacy at 10 years.

Predictor variables	Odds Ratio^a^ ^b^		p
	OR	95%CI	
**PPVT group at age 4**			0.0215
Low (bottom 15%)	1.55	1.07–2.25	
Middle-high group	ref.		
**PPVT group at age 6**			0.0485
Low (bottom 15%)	1.42	1.00–2.00	
Middle-high group	ref.		
**PPVT group at age 8**			<.0001
Low (bottom 15%)	2.2	1.59–3.03	
Middle-high group	ref.		
**Child Characteristics at age 4**			
**Ethnicity**			0.0003
ATSI^c^	2.94	1.65–5.26	
SC Non-ATSI	ref.		
**Who Am I (quintiles)**			<.0001
1 (lowest)	7.35	4.5–12.02	
2	3.84	2.38–6.19	
3	2.43	1.41–4.21	
4	1.31	0.76–2.28	
5 (highest)	ref.		
**Persistence (quintiles)**			0.2727
1 (lowest)	0.99	0.62–1.58	
2	1.29	0.83–2.02	
3	0.82	0.5–1.35	
4	1.02	0.62–1.68	
5 (highest)	ref.		
**Reactivity (quintiles)**			0.1146
1 (most reactive)	1.69	1.06–2.68	
2	1.72	1.1–2.69	
3	1.22	0.79–1.9	
4	1.39	0.89–2.18	
5	ref.		
**Maternal Characteristics at age 4**			
**Mother smoker**			0.1928
yes	1.25	0.89–1.76	
no	ref.		
**Maternal education**			0.5204
Year 12	1.02	0.7–1.47	
Year 11 or less	1.19	0.83–1.71	
University	ref.		
**Maternal consistency (quintiles)**			0.0131
1 (least consistent)	1.5	0.98–2.29	
2	1.13	0.75–1.7	
3	0.75	0.46–1.23	
4	0.84	0.55–1.3	
5	ref.		
**Family characteristics at age 4**			
**Family structure**			0.5918
Single mother family	1.14	0.71–1.83	
Other	ref.		
**Family income**			0.7115
under $600/week	1.47	0.77–2.84	
$600–999	1.36	0.83–2.23	
$1,000-$1,499	1.2	0.73–1.96	
$1,500-$1,999	1.32)	0.84–2.09	
$2,000 or more/week	ref.		
**Health care card**			0.1137
yes	1.40	0.92–2.13	
no	ref.		
**SEIFA disadvantage index (quintiles)**			0.7307
1 (lowest SEIFA)	0.89	0.53–1.51	
2	1.00	0.62–1.63	
3	0.79	0.47–1.32	
4	0.80	0.49–1.3	
5	ref.		
**Reads to child**			0.7095
no reading	1.39	0.64–3.02	
1–2 days/ week	1.20	0.85–1.70	
3–5 days/ week	1.1	0.83–1.47	
daily	ref.		

^a^Weighted

^b^95% Confidence Intervals

^c^Study child Aboriginal and/or Torres Strait Islander status

In the multivariate model three predictors exhibited substantial associations with subsequent low ARS at age 10: Low school readiness (WAI), Aboriginal and/or Torres Strait Islander status, and low PPVT at 8 years. Children in the lowest quintile for school readiness were at 7.35 the odds of children in the highest quintile of school readiness for low ARS at 10 years. Aboriginal and/or Torres Strait Islander children were at 2.94 the odds of non-Indigenous children for low ARS at age 10. Finally, children with low PPVT at 8 years were at 2.20 the odds for low ARS at 10 years, relative to children with middle-high PPVT at 8 years.

Moderate effects on low ARS at 10 years were observed with three more predictors: High temperamental reactivity, low PPVT at 4 years, and low PPVT at 6 years. Children in the most reactive quintile for temperamental reactivity at 4 years were at 1.69 the odds of children in the lowest quintile of temperamental reactivity for low ARS at 10 years. Children with low PPVT at 4 years were at 1.55 the odds for low ARS at 10 years, relative to children with middle-high PPVT at 4 years. Finally, children with low PPVT at 6 years were at 1.42 the odds for low ARS at 10 years, relative to children with middle-high PPVT at 6 years.

The remaining nine risk factors were not statistically significant in the multivariate model: Low maternal consistency, low family income, health care card, child not read to at home, maternal smoking, maternal education, family structure, temperamental persistence, and neighbourhood disadvantage.

### Sensitivity-specificity analysis

Odds ratios and statistically significant associations do not necessarily lead to clear predictive relationships. The fit of logistic regression models can be estimated via the area under the Receiver Operating Curve (ROC) statistic; Hanley and McNeil [48] recommend interpreting this as a measure of the discriminative utility of the model (that is, the probability that a randomly chosen child with low ARS at age 10 will be correctly rated, relative to a child that does not have low ARS at age 10). However this overall statistics does not measure the diagnostic utility of the model; to do so the researcher needs to establish a cut-point at which a child is diagnosed as a case or non-case (that is, at what probability do we predict a child will have low PPVT at age 8) [49, 50]. Loong [50] recommends presenting data in the form of a table, representing a trade-off between sensitivity and specificity, and between positive predictive value and negative predictive value.

Single binary predictors in a logistic regression model permit the calculation of sensitivity, specificity, positive predictive value and negative predictive value based on the presence or absence of that predictor. That is, when considering a single binary predictor, there is single binary cut-point for predicting cases from non-cases. When considering low PPVT at age 4 as a predictor of low ARS at age 10 (as in Table 6), children in the low PPVT group are at 3.43 the odds of children in the middle-high group for low ARS at age 10. However, this does not reveal how well PPVT at age 4 predicts ARS at age 10.

**Table 6:** Predicting literacy at age 10 from vocabulary at age 4.

	Actual literacy status age 10		
Language status at age 4	Low literacy at 10 years	Middle-high literacy at 10 years	Total
Low (bottom 15%)	114	247	361 predicted cases
Middle-high group	312	2351	2663 predicted non-cases
	416 actual cases	2598 actual non-cases	

In Table 6 we term children with low ARS at age 10 ‘cases’ and children who do not have low ARS at age 10 ‘non-cases’. Based on this model, the only means by which we can predict whether or not a child will have low ARS at age 10 is whether or not they have low PPVT at age 4. A prediction on this basis classifies all 361 of the children with low PPVT at age 4 to have low ARS at age 10. Table 7 also shows that 114 out of these 361 children actually have low ARS at age 10, resulting in a positive predictive value of 114/361 (31.5%). This identifies 114 of the 416 actual cases at age 10, giving the model a sensitivity of 114/416 (27.4%). Of the 2663 children predicted to be non-cases at age 10 based on having middle-high PPVT at age 4, 2351 were in fact non-cases, giving the model a negative predictive value of 2351/2663 (88.3%). The model identified 2351 out of 2598 non-cases, giving the model a specificity of 2351/2598 (90.4%).

**Table 7:** Sensitivity-Specificity of Multivariate Logistic Model at probability cut-off of 0.13.

	Actual literacy status age 10		
Language status at age 4	Low literacy at 10 years	Middle-high literacy at 10 years	Total
Low literacy at 10 years	212	575	787 predicted cases
Not Low literacy at 10 years	91	1438	1529 predicted non-cases
Total	303 actual cases	2013 actual non-cases	2316

However, when considering a multivariate model, information from different predictors can be combined and a variety of cut-points can be chosen. Thus, to select a cut-point requires judgements that concern the utility of the model. These judgements result in trade-offs between sensitivity and specificity and between positive predictive value and negative predictive value depending on the gravity (e.g. economic or ethical) of misclassification. A cut-off threshold of probability for predicting whether the study child will have low ARS at age 10 must be set by the investigator.

**Fig 3:**
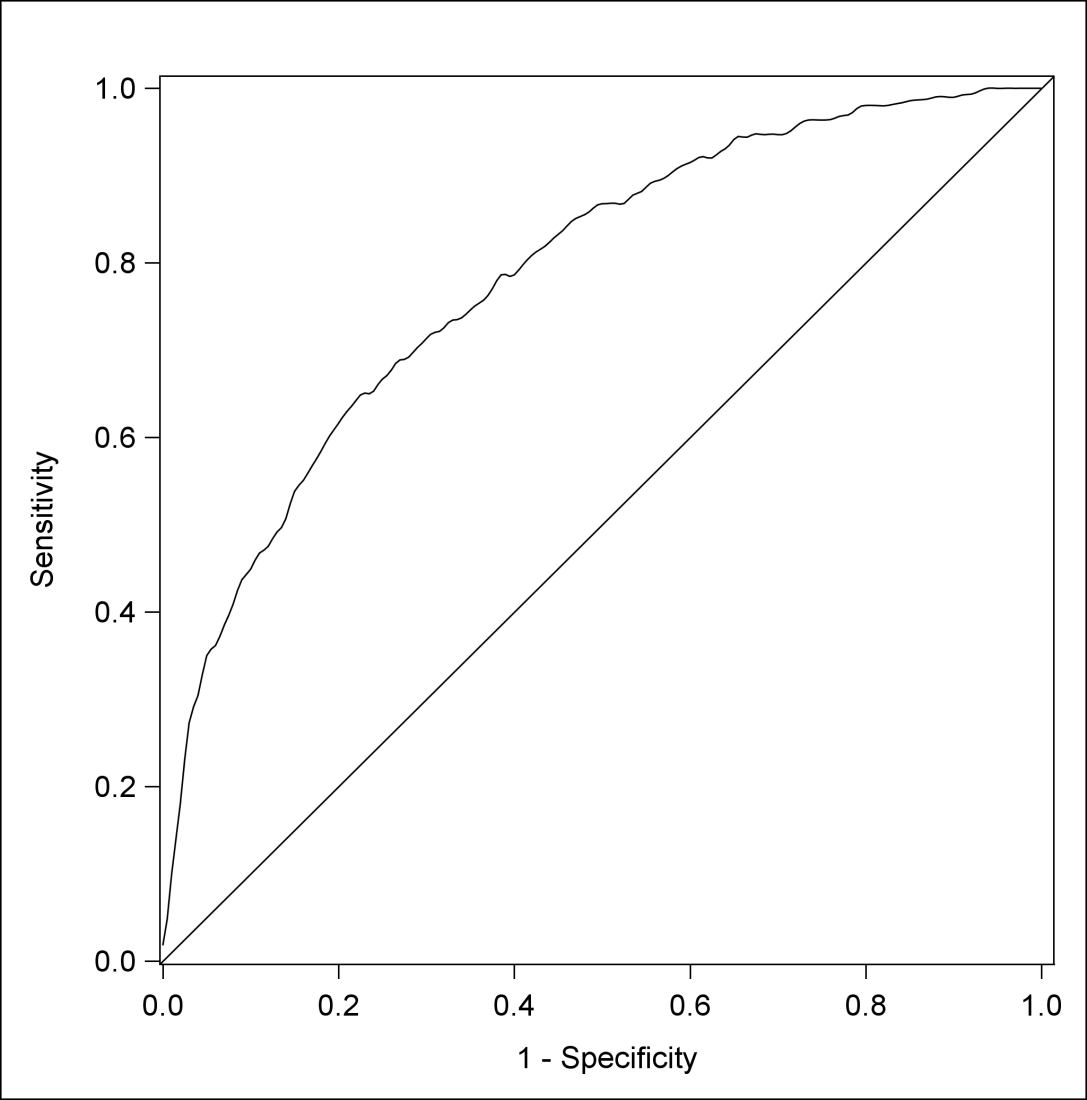
Sensitivity-Specificity Curve, Multivariate Logistic Model.

In the absence of clear utility guidelines for setting a criterion cut-off for our model, we examined the ROC curve (see Fig 3) and set a probability threshold of 0.13 to give an approximately equal level of sensitivity (0.700) and specificity (0.714). Hosmer and Lemeshow [51] would term this ‘acceptable’ discrimination.

There were 2316 children in the model at age 4 (Table 7). At the cut-point for equal sensitivity and specificity, the final multivariate model classified 787 children as having low ARS at age 10. However, by the time they were assessed at age 10, 303 children were actually found to have low ARS. In line with our criterion cut-off, the model predicted that 212 of these 303 children would have low ARS (sensitivity = 70.0%). This level of sensitivity also carried with it the classification of another 575 children into the low ARS group at age 10 when, in fact, these children did not have low ARS on examination. This model thus produces a positive predictive value of 26.9% (212/787).

Conversely, by the time the 2316 children were assessed at age 10, 2013 were actually found to have middle-high ARS. In line with our criterion cut-off, the model predicted that 1438 of these children would have middle-high ARS (sensitivity = 71.4%). This level of specificity also carried with it the classification of another 91 children as not having low ARS group at age 10 when, in fact, these children had low ARS on examination at age 10. This model thus produces a negative predictive value of 94.0% (1438/2316).

Other criterion could be chosen. For example, by choosing a stricter cut-off such as 0.05, sensitivity increases to 92.7% but decreases the specificity to 36.6%. Or, by relaxing the cut-off to 0.18 the sensitivity of our model decreases to 59.1% but specificity to increases to 81.6%. However, these adjustments all entail varying levels of positive and negative predictive power.

## Discussion

Understanding factors in early childhood that promote and impede children’s progress along the oral-literate continuum at school has long been a primary concern of educational policy. What typically happens to children as they age from 4 to 10 years in their progression from early and rapid vocabulary acquisition to their onward and early literacy performance? The aims of this study were to identify developmental patterns of vocabulary and literacy development in this age period, to assess associated predictors of early literacy in this age range, and estimate their predictive utility. The strength of this study resides in a well characterized sample with measures selected, calibrated and independently collected longitudinally on the same children from multiple informants.

### Developmental patterns of vocabulary and literacy

Six patterns of vocabulary development to onward early literacy were longitudinally observed across 4 biennial assessments from age 4 to 10. The most common pattern was a stable developmental pattern in which children commenced with, and then maintained, middle-high vocabulary ability that progressed to an outcome of middle-high literacy ability. This pattern fit 69% of children.

Collectively, another 30% of the children exhibited four change patterns. These comprised: 1) an improving pattern of vocabulary development towards onward literacy for 8% of children; 2) a declining pattern for another 10% of children; 3) a fluctuating low pattern for 2% of children; and, 4) a fluctuating middle-high group for 10% of the children.

One of the least common developmental patterns was a stable pattern comprising initial and subsequent persistent low vocabulary progressing to an outcome of low literacy abilities. Less than 1% (26/2792) of children in our study fit this pattern.

Our findings allow an early and rare look at the transition point from early vocabulary development to onward early school literacy. We demonstrate considerable positional instability in the vocabulary development of this cohort and model its relationship to literacy performance at age 10. The onward literacy performances of these children will become apparent as successive waves of data are gathered. At this point though, our findings are similar to those of Verhoeven et al who reported the association between early vocabulary and word decoding to be consistently significant but only weak [52].

In addressing this initial instability of literacy development, previous research in very young children has focussed principally upon the growth of literacy. Using both cross-sectional and longitudinal samples of young children and measuring their oral and reading vocabulary, these studies have demonstrated very rapid developmental growth of literacy in the epoch from pre-school to Year 4 [53–56]. In later years of schooling there is then some evidence to suggest that the average growth of literacy decreases as students move from the early grades to the last grade of middle school [57]. Such studies though are relatively silent about the longitudinal instability in within-individual levels of performance as children move (i.e. age) from pre-literacy to early literacy.

There is some research however that has highlighted developmental instability of literacy with longitudinal samples. Leppänen et al (2004) specifically investigated the variability in reading performances of pre-schoolers as they moved into early primary school [58]. They demonstrated that during the preschool year individual differences in reading grew larger and that this growth was faster among those who entered preschool with well-developed skills. However, during the first grade individual differences in reading then diminished with poor readers developing reading at a faster rate than good readers. In other words, there was decreasing variability between individuals of differing ability during the first grade. Our results, of course, provide a description of the initial literacy position of children in their first year and models how this is predicted by prior vocabulary growth.

While scant, other examples of developmental instability in literacy performance exist. Using a small sample of 187 children followed from Year 1 to Year 6 Phillips et al., 2002 [20], found a much higher probability than had previous studies, for children below average in first grade to be average in subsequent grades; a significant probability for average students to become above average, where none were documented previously; and an almost equal probability of above-average readers becoming average as remaining above average (Phillips et al, 2002; pp. 10–11 [20]). In an extensive examination of school attainment mobility using the 1958 and 1970 UK British Cohort Studies and the 2002 National Pupil Database, Feinstein (2004) documented “considerable shifts” in reading, maths, and key stage levels such that in the 1958 cohort, 35% of children in the bottom quartile had “escaped” to higher performance levels [59]. In a similar vein, using the Avon Longitudinal Study of Parents and Children (ALSPAC), Duckworth [60] observed both “considerable” stability and “substantial” movement in literacy performances of children 5 to 11 years of age. Finally, and echoing Leppänen el, longitudinal studies among children who commence their literacy careers with identified reading disability have also returned evidence that longitudinal stability of performance was poor, with the poorest stability for the low growth definition [61].

### Early predictors of later low literacy

In examining early predictors for low literacy ability at age 10 we identified three substantial contributors with adjusted effects meeting our criterion: Low school readiness at age 4, Aboriginal and/or Torres Strait Islander status and low vocabulary ability at age 8. High child temperamental reactivity had a moderate predictive relationship with low literacy at age 10 as did low Vocabulary at ages 4 and 6.

The highest risk for low literacy ability at age 10 was low school readiness as measured by the *Who Am I*? at age 4. The finding that pre-academic early copying and writing skills substantially overshadows low vocabulary ability as a risk factor for low literacy at age 10 is consistent with other studies that have shown that explicitly taught pre-academic skills that children bring to school make a major contribution to their academic progress at school [62]. In the face of this, and with regard to what the child is actually asked to perform on the WAI, it is plausible to assert the WAI measures some aspects of ‘school-readiness’. There are two points that we would make in this regard.

First, Sénéchal and LeFevre [63] have shown that children’s exposure to books and to parents’ reports of how frequently they *taught* (italics added) their children about reading and printing words were uncorrelated and showed distinct pathways into language and literacy: “…storybook reading was related to children’s receptive language development, whereas parents’ reports of teaching were related to children’s early literacy skills (p. 455)”. Our findings that a child’s pre-academic early copying and writing skills at age 4 substantially predict onward literacy at age 10 parallels these findings. It is also notable that in our model, neither maternal education nor income was predictive of onward low literacy. We would speculate that in the presence of a more proximal measure of direct inputs to the child’s early abilities (as measured by the WAI) both maternal education and family income—which are proxies for human capital and material resources—become non-significant.

Second, school readiness is a multidimensional construct. It comprises physical health and wellbeing, social competence, emotional maturity, language and cognitive development, and communication skills and general knowledge. The WAI measures more multiple dimensions of school readiness relative to the PPVT, which measures one dimension (semantics, language, or verbal cognitive ability depending on perspective). In total population studies of Australian 5 year olds using the Australian Early Development Index (EDI), Brinkman et al. [64] demonstrated that children’s scores in the language/cognitive skills domain and the communication skills/general knowledge domain of the EDI at age 5 correlated most strongly with national standardised measures of reading at age 10 in Grade 5 (0.40 and 0.33 respectively). Critically though, vulnerability in any one domain of the EDI at age 5 increased the risk of scoring in the bottom 20% of these standardised national measures of Reading in Grades 3, 5 and 7. This provides necessary evidence of the multidimensionality of school readiness.

Our study shows that measuring multiple dimensions of school readiness with the WAI at age 4 in the year before the start of formal school, and one year earlier than EDI, identifies vulnerability for literacy measured 6 years later when the children are age 10. Children’s literacy development begins long before the start of formal school and the magnitude of risk associated with low school readiness at age 4 for low literacy at age 10 provides strong evidence of the importance of the preschool period for reducing inequalities in literacy acquisition at school through the provision of developmentally enriched opportunities and programs.

The second substantial risk for low literacy performance was carried by children of Aboriginal and Torres Strait Islander descent. At the outset it is important to distinguish these children from the Australian population of Indigenous children: The LSAC sample excluded children living in remote areas of Australia and was not designed to representatively sample Indigenous children nor procure sufficient numbers of Indigenous children from which to derive stable population estimates (see Baxter, 2012 [65]). The Wave 1 LSAC sample principally represents urban and regional Indigenous children, the majority (90%) of whose mothers spoke English as their primary language. These children lived with two parents (59%) or with a sole parent (with or without another adult carer) (39%).

Differential attrition has affected this subsample over the four waves. One hundred children of Aboriginal and Torres Strait Islander descent (n = 100) were available for our analysis. In this sample, 98% of the mothers reported that English was their primary language. This leaves 2 of our Aboriginal and Torres Strait Islander children effectively coded as having a “Non-English speaking mother at Wave 1.” The higher risk for poor performance on the ARS estimated for Aboriginal and Torres Strait Islander status in our sample is not likely to be attributable in large part to second language use within the home.

What then more plausibly explains the higher risk for poor onward literacy among the Aboriginal and Torres Strait Islander children in this sample? The challenging and confronting circumstances that have beset politicians, administrators and practitioners in improving the life prospects and capabilities of Australia’s Indigenous peoples are well described [66, 67]. Our finding that Indigenous status predicts onward literacy at age 10 in this relatively high-functioning sub-sample of Indigenous children is both confirmatory of this circumstance and noteworthy. It is noteworthy because the final model adjusts for pre-academic readiness and several other potential influences on the onward development of literacy in these children. Nonetheless, Indigenous status continues to mark onward lower achieved literacy. The basis for this increasingly implicates diminished expectations and opportunities for these children with concomitant lower proportionate developmental investments to lift their capability above their non-Indigenous counterparts [66, 68].

The third substantial predictor of literacy at age 10 was vocabulary ability at age 8. While the age 4 and age 6 measures of vocabulary showed moderate and progressively weaker associations with literacy relative to the more proximal age 8 measure of vocabulary, all retained significance in the multivariate model.

In line with Sénéchal and LeFevre’s (2002) [63] observation that storybook reading was related to children’s receptive language development, our measure of parental book reading, in the presence of direct measures of the children’s (receptive) vocabulary (PPVT), was not significant in the multivariate model. Vocabulary ability predicts literacy but its likely contribution to literacy acquisition is through the enablement, support, and expansion of receptive language. In this regard our findings support those of Verhoven et al who observed the vocabulary association with reading comprehension to be much stronger than with word decoding (Verhoven et al, 2011, p. 19).

We have previously documented the high variability in rates of children’s vocabulary growth and the volatility in their positional change over time [21, 22]. Our findings here permit a view of the general developmental “bridging” between children’s initial levels and onward rates of vocabulary development and their subsequent literacy level at age 10. While vocabulary proficiency at each age from 4 to 8 is predictive of onward literacy, just as the high variability in children’s vocabulary performance over time produced very poor utility in predicting their onward vocabulary performance (see Christensen, et al, 2014 [22]), the onward prediction of literacy remains equally challenging.

The final remaining predictor of any significance was temperamental reactivity. In our model early temperament predicts onward low literacy—but only in so far as this pertains to reactivity (sitting still, paying attention, etc) rather than persistence (focusing and working on one thing). The effect of temperament on language development has a mixed career with some researchers finding higher rates of temperamental problems in children with language delay [69–72] and others not [73, 74]. In contrast, there is evidence of some effects of temperament on academic achievement of young children [75, 76]. Coplan et al. (1999) [77] studied temperamental reactivity and showed that preschool children with greater attention spans, lower activity levels, and lower negative emotionality at the beginning of the school year performed significantly better on direct assessments of literacy and numeracy skills at the end of the school year.

### Implications of low predictive utility

The findings of low predictive utility between vocabulary development, an important marker of language development, and onward literacy ability reinforce a general view that the early selection and exclusive targeting of children on the basis of risk indicators will miss a large proportion of children who are not performing low at the point of selection but who will go on to sustain low outcomes at later points. Such predictions will at the same time, deliver into the initial selection of low performing children, a substantial number who will developmentally improve without intervention.

This means that although we identified substantial risks with adjusted odds ratios of greater than 2.0, the analysis of predictive utility (sensitivity, specificity, positive predictive value and negative predictive value) indicates that these risk factors do not function well to predict who will and will not have low literacy ability at age 10. We would note that researchers often conflate odds ratios with predictive utility, in that interventions are discussed in light of odds ratios similar to those in this paper, without giving consideration to the utility of the predictive relationship. We would encourage other researchers in the field to consider this distinction more carefully.

We acknowledge that our models use a statistical, not a diagnostic or clinical definition, of low vocabulary and low literacy status. With this said though, our presentation of the estimates of poor predictive utility does not carry with it the intention of dismissing the importance of demonstrated significant predictors of literacy nor a desire to undermine the pursuit and implementation of interventions to address literacy in young children. Low early school readiness, Indigenous status, and low prior vocabulary development heighten the risks of progression towards low literacy competency.

However, what our estimates of clinical utility do illustrate is “about” how hard it is to predict the literacy status of 10 year olds from a reasonable set of well measured variables selected with the theoretical and practical intent to enable this. The findings show that ‘late’ identification is inevitable for children whose low performance is not evident earlier in development. In this study, 10.2% of children showed a declining pattern of development. That is, they shifted from adequate performance at an earlier age to low performance at a subsequent age. Furthermore, 53% (202/381) of children with low literacy at age 10 were not in the low performance groups for language at ages 4, 6, and 8. For these children, identification at any earlier age was not possible. This reflects a developmental truism rather than failure of the monitoring system to detect low performance at an earlier age. This is an important message for policy makers, practitioners and parents. These observations are relevant for prevention scientists and practitioners alike.

For prevention scientists seeking to implement randomised trials, there are clear hazards in both selection and trial assignment and in obtaining the necessary power to make estimates of effects. Where children are selected at an early point on the basis of low performance and assigned to treatment and no-treatment conditions the observed and typical variation as seen here may in all likelihood contribute to a pattern of mixed results across studies and weak effects in controlled trials (see for example Duff et al, 2014 [78]).

Where unselected samples are procured for intervention the high levels of developmental variability seen here will require careful scientific consideration of how to appropriately power and gear the sample size in order to differentiate the treatment effect of interest from the existing and high level of typical developmental variability. In intervention terms, the treatment “signal” will need to overcome a substantial level of typical developmental “noise” in order to be detected. For intervention scientists, the design choices here typically move across interacting fronts: The selection of sensitive child development measures that are calibrated with precision and, the specification of sample sizes with the power to detect the effect of the intervention. Our study serves as a reminder that if variability is part of the natural history of childhood language development, intervention scientists need to ensure they allow adequate sample to detect change. In our observational longitudinal study the measures used are suitable for use in large unselected samples. They have known and reasonable properties and are also used in school and health settings. Even with the presence of a large sample, and reasonable measures, our findings point to the challenges that intervention scientists face in the design of studies that test intervention efficacy.

For those in policy and practice there are several pragmatic implications from these findings on the poor predictive utility of the multivariate and the change patterns that were observed. How do services (health, welfare, education) deliver preventive and treatment opportunities that take account of the risk profile of children in the face of their poor predictive utility?

The findings here continue to encourage the adoption of methods of delivering services that are proportionately universal. This however, requires developing a policy platform of universal services available to all children irrespective of their risk status while incorporating the capacity to select and reach the more vulnerable. This is more than “repackaging” existing services. The service arrangement must allow for better documenting of a child’s developmental growth: In other words, an observation at a single point in time will not be useful relative to small annual or biennial measures in this period. Service arrangements will need to provide a relatively “light touch”, but universal capacity to measure children and document their growth over time to more appropriately detect those children that maintain stable low patterns, fluctuating low patters, and declining low patterns. This would permit better precision in targeting developmental prevention and intervention efforts to those that are demonstrating need [79].

As well as this though, a particular tension in the implementation of policies and practices based on proportionate universalism rests with the propensity of the targeting component to focus on treatment (early or otherwise) to the exclusion of more robust efforts at altering barriers and reaching participants for prevention opportunities in the first place. Treatment will certainly be needed for those identified in need of it—but the gap in the targeted component tends to be in the failure to alter and address barriers for inclusion of higher risk children in prevention opportunities. How do we deliver effective developmental opportunities to those more in need? This remains a vexing and persistent challenge and intensification of this opportunity represents the “proportionate” effort required in policy and service implementation.

In summary, for those wishing to reduce the risks for low literacy in the early preschool period, our findings suggest interventions in the preschool periods should provide broad-based developmental opportunities for children with interventions that improve school readiness—this multidimensional construct includes self-regulation (e.g., inhibitory control/low reactivity) and general language enrichment. This should be programmatically delivered with multiple ‘touches’ across development and ‘light touches’ early in development because of the inevitability of over-servicing and be based on proportionate universalism that pays special attention to reducing barriers to participation for vulnerable groups (e.g., in our model, Aboriginal children).

Our study has limitations.

First, we lack a measure of non-verbal ability. There are strong arguments in favour of having such a measure (see Rice and Hoffman, 2014 [80]). While it could be claimed that the WAI has items that tap some non-verbal capacities, it is not a molar measure of these. Second, our modelling only permits examination of the predictive utility of a single language component—semantics, as measured by receptive vocabulary. While vocabulary is certainly a key language outcome and predictor of literacy it is not a surrogate of all dimensions of language change over time [80]. Third, it could be argued that interest in and concerns about low literacy and its prediction in the period from 4 to 10 focuses “too late” in the language development trajectory. At best, this is only somewhat true. In reality, teachers and practitioners regularly see children in this period of development for determination of their developmental status and their language and reading capacities. Our findings are informative of the patterns, risks, and predictive setting that confront teachers and practitioners. What happens in language development from 4 to 8 years and how this connects with literacy at 10 is also important in terms of onward lifecourse outcomes and has a direct bearing on how early childhood development opportunities and early childhood education are configured.

### Future research

In this study, we modelled the effect of multivariately adjusted single risk exposures (e.g., low school readiness) in early childhood on low literacy in middle childhood and discussed the implications of this approach to multi-level prevention and intervention policies and practice. In a subsequent study, we will investigate the effect of multiple risk exposures in early childhood on low literacy in late childhood. This will allow us to identify children with disproportionate exposure to multiple risks in early childhood and to determine the predictive utility of a multiple risk index for low literacy in late childhood. To achieve the necessary statistical power to conduct these sub-group analyses, we will expand the current analytic sample to include the the younger LSAC age cohort, that was out of scope for the current study. Combining the full LSAC sample of 10,000 children will be necessary to have adequate power to investigate risk and protective factors for declining and improving change patterns.

In the future, we will be able to assess trajectories in language and literacy development in the LSAC using a direct assessment measure of children’s literacy. In Australia, the National Assessment Program—Language and Literacy (NAPLAN) [81] assesses all children in reading, writing, language conventions and numeracy in Years 3, 5, 7 and 9. Data linkage methods will be used to link survey data to NAPLAN data. We plan to use LSAC survey data and NAPLAN data to further understand patterns in children’s developmental progress in language and literacy and school achievement.

## References

[1] Schoon I, Parsons S, Rush R, Law J. Childhood language skills and adult literacy: A 29-year follow-up study. Pediatrics. 2010;125(3):459–66. 10.1542/peds.2008-2111 20142287

[2] Johnson CJ, Beitchman JH, Brownlie EB. Twenty-Year Follow-Up of Children With and Without Speech-Language Impairments: Family, Educational, Occupational, and Quality of Life Outcomes. American Journal Of Speech-Language Pathology. 2010;19(1):51–65. 10.1044/1058-0360(2009/08-0083) 19644128

[3] Australian Curriculum Assessment and Reporting Authority (ACARA). 2013. Available: http://www.australiancurriculum.edu.au/. Accessed 8 February 2013.

[4] Neuman SB, Dickinson DK, editors. Handbook of early literacy research. New York: The Guilford Press; 2011.

[5] Pianta RC, editor. Handbook of early childhood education. New York: The Guilford Press; 2012.

[6] Hattie J. Visible learning: A synthesis of over 800 meta-analyses relating to achievement. New York: Routledge.

[7] Catts HW, Fey ME, Tomblin JB, Zhang X. A longitudinal investigation of reading outcomes in children with language impairments. Journal of Speech, Language and Hearing Research. 2002;45:1142–57. 10.1044/1092-4388(2002/093) 12546484

[8] Brownlie EB, Beitchman JH, Escobar M, Young A, Atkinson L, Johnson C, et al Early language impairment and young adult delinquent and aggressive behavior. Journal of Abnormal Child Psychology. 2004;32(4):453–67. 15305549 10.1023/b:jacp.0000030297.91759.74

[9] Law J, Rush R, Schoon I, Parsons S. Modeling developmental language difficulties from school entry into adulthood: Literacy, mental health, and employment outcomes. Journal of Speech, Language, and Hearing Research. 2009;52:1401–16. 10.1044/1092-4388(2009/08-0142) 19951922

[10] NICHD Early Child Care Research Network. Pathways to reading: The role of oral language in the transition to reading. Developmental Psychology. 2005;41(2):428–42. 10.1037/0012-1649.41.2.428 15769197

[11] United Nations Educational Scientific and Cultural Organisation. Education for All Global Monitoring Report 2006. Available: http://www.unesco.org/education/GMR2006/full/chapt2_eng.pdf. Accessed 18 February 2013.

[12] Australian Institute of Health and Welfare. Headline indicators for children's health, development and wellbeing. Cat no. PHE 144. Canberra: AIHW, 2011.

[13] Rose J. Independent review of the teaching of early reading: Final report. London, UK: Department for Education and Skills; 2006 Available: http://webarchive.nationalarchives.gov.uk/20100526143644/http:/standards.dcsf.gov.uk/phonics/report.pdf. Accessed 10 December 2014.

[14] Feinstein L, Duckworth K. Development in the early years: Its importance for school performance and adult outcomes. London: Institute of Education; 2006 Available: http://eprints.ioe.ac.uk/5970/1/Feinstein2006Development.pdf. Accessed 13 February 2013.

[15] Gough P, Tunmer W. Decoding, reading and reading disability. Remedial and Special Education. 1986;7(1):6–10.

[16] Powell D, Diamond K. Promoting early literacy and language development In: Pianta R, editor. Handbook of Early Childhood Education. New York: The Guilford Press; 2012 p. 194–216.

[17] Bishop DVM, Snowling MJ. Developmental Dyslexia and Specific Language Impairment: Same or Different? Psychological Bulletin. 2004;130(6):858–86. 15535741 10.1037/0033-2909.130.6.858

[18] Judge S, Bell SM. Reading achievement trajectories for students with learning disabilities during the elementary school years. Reading and Writing Quarterly. 2011;27(1–2):153–78. 10.1080/10573569.2011.532722

[19] Parrila R, Aunola K, Leskinen E, Nurmi J, Kirby J. Development of individual differences in reading: Results from longitudinal studies in English and Finnish. Journal of Educational Psychology. 2005;97(3):299–319. 10.1037/0022-0663.97.3.299

[20] Phillips L, Norris S, Osmond W, Maynard A. Relative reading achievement: A longitudinal study of 187 children from first through sixth grade. Journal of Educational Psychology. 2002;94:3–13. 10.1037/0022-0663.94.1.3

[21] Taylor C, Christensen D, Lawrence D, Mitrou F, Zubrick S. Risk factors for children’s receptive vocabulary development from four to eight years in the Longitudinal Study of Australian Children. PLOS ONE. 2013;8(9):e73046 10.1371/journal.pone.0073046 24039856 PMC3770643

[22] Christensen D, Zubrick S, Lawrence D, Taylor C. Risk factors for low receptive vocabulary abilities in the preschool and early school years in the Longitudinal Study of Australian Children. PLOS ONE. 2014;7(9):e101476 10.1371/journal.pone.0101476 PMC4079336 24988308

[23] Soloff C, Lawrence D, Johnstone R. LSAC Sample Design. Melbourne: Australian Institute of Family Studies, 2005. (Technical Paper No. 1).

[24] Soloff C, Lawrence D, Misson S, Johnstone R. Wave 1 weighting and non-response. Melbourne: Australian Institute of Family Studies, 2006 (Technical Paper No. 3). 3 8 2012. Report No.

[25] Misson S, Sipthorp M. Wave 2 weighting and non-response (Technical Paper No.5). Melbourne: Australian Institute of Family Studies, 2007.

[26] Rothman S. An Australian version of the Adapted PPVT-lll for use in research. Melbourne: Australian Council for Educational Research, 2003.

[27] Dunn LM, Dunn LM, Williams KT. Peabody Picture Vocabulary Test-III. Circle Pines, MN: American Guidance Service; 1997.

[28] Kim S, Camilli G. An item response theory approach to longitudinal analysis with application to summer setback in preschool language/literacy. Large-scale assessments in Education. 2014;2(1). 10.1186/2196-0739-2-1

[29] Rothman S. The LSAC Academic Rating Scale Score. Melbourne, Victoria: Australian Council for Educational Research, 2009.

[30] Rock DA, Pollack JM. Early Childhood Longitudinal Study, Kindergarten Class of 1998–99 (ECLS-K), Psychometric report for kindergarten through First Grade: (NCES 2002–05). National Center for Education Statistics, U.S. Department of Education Washington, DC; 2002.

[31] Pollack JM, Rock DA, Weiss MJ, Atkins-Burnett S. Early Childhood Longitudinal Study, Kindergarten Class of 1998–99 (ECLS-K), Psychometric Report for the Third Grade: (NCES 2005–62). National Center for Education Statistics, Institute of Education Sciences, U.S. Department of Education Washington DC; 2005.

[32] Bronfenbrenner U. The Ecology of Human Development. Cambridge, MA: Harvard University Press; 1979.

[33] Bronfenbrenner U. Making human beings human: Bioecological perspectives on human development. Thousand Oaks, CA: Sage Publications; 2005.

[34] de Lemos M, Doig B. Who am I? Developmental Assessment Manual. Melbourne: Australian Council for Education Research; 1999.

[35] Prior M, Bavin E, Ong B. Predictors of school readiness in five- to six-year-old children from an Australian longitudinal community sample. Educational Psychology: An international Journal of Experimental Educational Psychology. 2013;31(1):3–16.

[36] Rothman S. Report on Adapted PPVT-III and Who Am I? Melbourne: Australian Council for Educational Research, 2005. (Data issues paper No. 2).

[37] Sanson A, Prior M, Oberklaid F, Garino E, Sewell J. The structure of infant temperament: Factor analysis of the Revised Infant Temperament Questionnaire. Infant Behavior & Development. 1987;10:97–104.

[38] Strazdens L, Shipley M, Broom D. What does family-friendly really mean? Wellbeing, time, and the quality of parents' jobs. Australian Bulletin of Labour. 2007;33(2):202–25.

[39] Strazdens L, Shipley M, Clements M, O'Brien L, Broom D. Job quality and inequality: Parents' jobs and children's emotional and behavioral difficulties. Social Science and Medicine. 2010;70:2052–60. 10.1016/j.socscimed.2010.02.041 20382458

[40] Lawrence D, Mitrou F, Zubrick S. Non-specific psychological distress, smoking status and smoking cessation: United States National Health Interview Survey 2005. BMC Public Health. 2011;11(256):256 10.1186/1471-2458-11-256 21513510 PMC3107796

[41] Zubrick S, Smith G, Nicholson J, Sanson A, Jackiewitz T, & the LSAC Research Consortium. Parenting and families in Australia: Social Research Policy Paper No. 34. Canberra: Australian Government Department of Families, Housing, Community Services and Indigenous Affairs, 2008.

[42] Zubrick S, Lucas N, Westrupp E, Nicholson J. Parenting measures in the Longitudinal Study of Australian Children: Construct validity and measurement quality, Waves 1 to 4. Canberra: Department of Social Services, 2014.

[43] ABS. Information paper—Census of Population and Housing, Socio-Economic Indexes for Areas, Australia 2001. Canberra: Australian Bureau of Statistics, 2003.

[44] SAS Institute Inc. SAS for Windows Version 9.3. Cary, NC: SAS Institute Inc.,; 2002–2010.

[45] Haddock CK, Rindskopf D, Shadish WR. Using Odds Ratios as Effect Sizes for Meta-Analysis of Dichotomous Data: A Primer on Methods and Issues. Psychological Methods. 1998;3(3):339–53.

[46] Allen J, Le H. An Additional Measure of Overall Effect Size for Logistic Regression Models. Journal of Educational and Behavioral Statistics. 2008;33(4):416–41.

[47] Valentine JC, Cooper H. Effect size substantive interpretation guidelines: Issues in the interpretation of effect sizes. Washington, DC: What Works Clearinghouse; 2003.

[48] Hanley JA, McNeil BJ. The meaning and use of the area under a receiver operating characteristic (ROC) curve. Radiology. 1982;143(1):29–36. Epub 1982/04/01. . 7063747 10.1148/radiology.143.1.7063747

[49] Altman DG, Bland JM. Diagnostic tests 3: receiver operating characteristic plots. British Medical Journal. 1994;309(6948):188 8044101 10.1136/bmj.309.6948.188 PMC2540706

[50] Loong T-w. Understanding Sensitivity And Specificity With The Right Side Of The Brain. BMJ: British Medical Journal. 2003;327(7417):716–9. 14512479 10.1136/bmj.327.7417.716 PMC200804

[51] Hosmer DW, Lemeshow S. Applied logistic regression. New York: Wiley Interscience; 1989.

[52] Verhoeven L, van Leeuwe J, Vermeer A. Vocabulary Growth and Reading Development across the Elementary School Years. Scientific Studies of Reading. 2011;15(1):8–25. 10.1080/10888438.2011.536125

[53] Lesaux NK, Rupp AA, Siegel LS. Growth in reading skills of children from diverse linguistic backgrounds: Findings from a 5-year longitudinal study. Journal of Educational Psychology. 2007;99(4):821–34. 10.1037/0022-0663.99.4.821

[54] White TG, Graves MF, Slater WH. Growth of reading vocabulary in diverse elementary schools: Decoding and word meaning. Journal of Educational Psychology. 1990;82(2):281–90. 10.1037/0022-0663.82.2.281

[55] Dale E, O'Rourke J. The living word vocabulary. Chicago: World Book/Childcraft International; 1981.

[56] Biemiller A, Slonim N. Estimating root word vocabulary growth in normative and advantaged populations: Evidence for a common sequence of vocabulary acquisition. Journal of Educational Psychology. 2001;93(3):498–520. 10.1037/0022-0663.93.3.498

[57] Dadey N, Briggs DC. A meta-analysis of growth trends from vertically scaled assessments. Practica; Asessessment, Research and Evaluation. 2012;17(14). http://pareonline.net/getvn.asp?v=17&n=14.

[58] Leppanen U, Niemi P, Aunola K, Nurmi J-E. Development of reading skills among preschool and primary school pupils. Reading Research Quarterly. 2004;39(1):72–93. 10.1598/RRQ.39.1.5

[59] Feinstein L. Mobility in Pupils' Cognitive Attainment During School Life. Oxford Review of Economic Policy. 2004;20(2):213–29. 10.1093/oxrep/grh012

[60] Duckworth K. What role for the three Rs? Progress and attainment during primary school: The Wider benefits of Learning Research Report Series No. 23; 2007. Available: http://eprints.ioe.ac.uk/5963/1/Duckworth2007what.pdf. Accessed 6 January 2015.

[61] Brown Waesche JS, Schatschneider C, Maner JK, Ahmed Y, Wagner RK. Examining Agreement and Longitudinal Stability Among Traditional and Response-to-Intervention-Based Definitions of Reading Disability Using the Affected-Status Agreement Statistic. Journal of learning disabilities. 2011;44(3):296–307. 10.1177/0022219410392048 21252372 PMC3248271

[62] Thomas E. Readiness to learn at school among five-year-old children in Canada: Statistics Canada; 2006. Available: http://www.statcan.gc.ca/pub/89-599-m/89-599-m2006004-eng.pdf. Accessed 25 June 2013.

[63] Senechal M, LeFevre J. Parental involvement in the development of children's reading skill: A five-year longitudinal study. Child Development. 2002;73(2):445–60. 11949902 10.1111/1467-8624.00417

[64] Brinkman S, Silburn S, Lawrence D, Goldfeld S, Sayers M, Oberklaid F. Investigating the Validity of the Australian Early Development Index. Early Education & Development. 2007;18(3):427–51. 10.1080/10409280701610812

[65] Baxter J. The family circumstances and wellbeing of Indigenous and non-Indigenous children In LSAC Annual statistical report 2012. Australian Institute of Family Studies, Melbourne 2013.

[66] Zubrick S, Silburn S, Lawrence D, Shepherd C, Mitrou F, DeMaio J, et al Population capability and human development models: Policy changes to improve the lives of Australian Aboriginal children and families In: Robinson G, Eickelkamp U, Goodnow J, Katz I, editors. Contexts of child development: Culture, policy and interventio. Darwin and Sydney: Charles Darwin University Press; 2008 p. 59–72.

[67] Mitrou F, Cooke M, Lawrence D, Povah D, Mobilia E, Guimond E, et al Gaps in Indigenous disadvantage not closing: a census cohort study of social determinants of health in Australia, Canada, and New Zealand from 1981–2006. BMC Public Health. 2014;14(201). 10.1186/1471-2458-14-201 PMC3937433 24568143

[68] Shepherd C, Li J, Zubrick S. Social gradients in Indigenous health in Australia. American Journal of Public Health. 2011;102(1):107–17. 10.2105/AJPH.2011.300354 22095336 PMC3490556

[69] Caulfield MB, Fischel J, DeBaryshe BD, Whitehurst GJ. Behavioral correlates of developmental expressive language disorder. Journal of Abnormal Child Psychology. 1989;17:187–201. 2745899 10.1007/BF00913793

[70] Carson DK, Klee T, Perry CK, Muskina G, Donaghy T. Comparisons of children with delayed and normal language at 24 months of age on measures of behavioral difficulties, social and cognitive development. Infant Mental Health Journal. 1998;19(1):59–75.

[71] Irwin JR, Carter AS, Briggs-Gowan MJ. The social-emotional development of "late-talking" toddlers. Journal Of The American Academy Of Child And Adolescent Psychiatry. 2002;41(11):1324–32. 12410075 10.1097/00004583-200211000-00014

[72] Paul R, James DF. Language delay and parental perceptions. Journal of the American Acadamy of Child and Adolescent Psychiatry. 1990;29(4):669–70. 10.1097/00004583-199007000-00030 2387808

[73] Rescorla L, Achenbach TM. Use of the Language Development Survey (LDS) in a national probability sample of children 18 to 35 months old. Journal of Speech, Language and Hearing Research. 2002;45(4):733–43. 10.1044/1092-4388(2002/059) 12199403

[74] Zubrick S, Taylor C, Rice M, Slegers D. Late language emergence at 24 months: An epidemiological study of prevalence, predictors and covariates. Journal of Speech, Language, and Hearing Research. 2007;50:1562–92. 18055773 10.1044/1092-4388(2007/106) PMC3521638

[75] Maziade M, Côté R, Boutin P, Boudreault M, Thivierge J. The effect of temperament on longitudinal academic achievement in primary school. Journal of the American Academy of Child Psychiatry. 1986;25(5):692–6. 3760419 10.1016/s0002-7138(09)60296-x

[76] Vitiello V, Moas O, Henderson H, Greenfield D, Munis P. Goodness of fit between children and classrooms: Effects of child temperament and preschool classroom quality on achievement trajectories. Early Education and Development [Internet]. 2012; 23(3):[302–22 pp.].

[77] Coplan R, Barber A, Lagace´-Se´guin D. The role of child temperament as a predictor of early literacy and numeracy skills in preschoolers. Early Childhood Research Quarterly. 1999;14:537–53.

[78] Duff F, Reen G, Plunkett K, Nation K. Do infant vocabulary skills predict school-age language and literacy outcomes? Journal of Child Psychology and Psychiatry. 2015:n/a–n/a. 10.1111/jcpp.12378 PMC4674965 25557322

[79] National Collaborating Centre for Determinants of Health. Let’s talk: Universal and targeted approaches to health equity Antigonish, NS: National Collaborating Centre for Determinants of Health, St. Francis Xavier University; 2013. Available: //nccdh.ca/images/uploads/Lets_Talk_Health_Equity_English.pdf. Accessed 15 January 2014.

[80] Rice M, Hoffman L. Predicting Vocabulary Growth in Children with and without Specific Language Impairment (SLI): A Longitudinal Study from 2 ½ to 21 Years of Age. Journal of Speech, Language, and Hearing Research. 2015 10.1044/2015_JSLHR-L-14-0150 PMC4398600 25611623

[81] Gordon C, Walpole I, Zubrick SR, Bower C. Population screening for cystic fibrosis: Knowledge and emotional consequences 18 months later. American Journal of Medical Genetics. 2003;120A:199–208. 12833400 10.1002/ajmg.a.20259

